# COVID-19 and Its Ophthalmic Manifestations: A Literature Review

**DOI:** 10.7759/cureus.55571

**Published:** 2024-03-05

**Authors:** Neal T Patel, Alexander Martinek, Raheel Shaikh, Payal Kahar, Deepesh Khanna

**Affiliations:** 1 Department of Foundational Sciences, Dr. Kiran C. Patel College of Osteopathic Medicine Nova Southeastern University, Fort Lauderdale, USA; 2 Department of Foundational Sciences, Morehouse School of Medicine, Atlanta, USA; 3 Department of Medicine, Dr. Kiran C. Patel College of Osteopathic Medicine Nova Southeastern University, Fort Lauderdale, USA; 4 Department of Health Sciences, Florida Gulf Coast University, Fort Myers, USA

**Keywords:** angiotensin-converting enzyme 2 (ace2), ocular, episcleritis, ophthalmoparesis, infectious conjunctivitis, viral conjunctivitis, ophthalmic complications, covid-19, coronavirus, sars-cov-2

## Abstract

Severe acute respiratory syndrome coronavirus 2 (SARS-CoV-2) is a novel coronavirus discovered in late 2019 in Wuhan, Hubei Province, China. The virus has now developed into a full-scale global pandemic affecting hundreds of millions of people to date. A majority of cases present with nonspecific acute upper respiratory symptoms. A wide range of systemic symptoms has been reported, with some patients presenting with nonspecific extrapulmonary symptoms. Recently, there has been an increased association of COVID-19-positive patients presenting with ocular symptoms. As an increasing number of patients present with ophthalmic manifestations, recognizing these visual symptoms is of utmost importance. Some patients may present with ocular symptoms as the first indication of COVID-19 infection; quickly isolating and starting treatment can aid in stopping the spread of this novel coronavirus. This review will describe the current epidemiology and pathophysiology of SARS-CoV-2, emphasizing the ophthalmic manifestations and their clinical course progression. Further, we will be reporting on the growing number of rare ocular manifestations that have occurred in some COVID-19-positive patients, along with the route of transmission, specific manifestations, and the treatment methods for both these pulmonary and extrapulmonary symptoms, specifically the ocular manifestations.

## Introduction and background

In 2019, the novel severe acute respiratory syndrome coronavirus 2 (SARS-CoV-2) virus emerged in Wuhan, China, as a new, easily transmissible pathogen leading to severe acute upper respiratory symptoms that have taken millions of lives [1]. SARS-CoV-2 has quickly spread globally, leading to an international pandemic [2, 3]. As of Jan. 23, 2024, there have been 702,181,714 cases reported globally, and of that, a total of 6,972,220 people have passed away from the virus, according to the Johns Hopkins University Coronavirus Resource Center [4]. The United States alone accounts for 110,660,955 of those cases and 1,193,042 of those deaths [4]. The disease has been termed "COVID-19" by the World Health Organization. The average incubation duration is between five and 14 days long [5]. 

The main symptoms exhibited in those affected by COVID-19 are similar to those exhibited in patients with some other form of pneumonia: cough, fever, fatigue, headache, and myalgia are among those most commonly observed [1, 6]. The virus also leads to alveolar damage diffusely which reduces oxygen saturation. Seriously ill patients can also suffer from cytokine storm as a response by the body in an attempt to eliminate the virus. However, an increasing number of patients have begun to present with various other systemic symptoms and signs such as neurological, hepatic, and ophthalmic [7-9].

The most common ocular manifestations that have been noted are episcleritis and keratoconjunctivitis and inflammation of the sclera and conjunctiva of the eye, respectively. A study conducted by Zhou et al. [10] examined postmortem eyes and surgical specimens of COVID-19-positive patients. Immunohistochemical assays were performed on each sample, revealing angiotensin-converting enzyme 2 (ACE2) receptors are expressed in the conjunctiva, cornea, and limbus surface of the eye. The findings presented in this paper emphasize the importance of the eye as a reservoir for COVID-19 and how transmission can occur without proper eye and hand care [10, 11]. The ACE 2 receptor is used by the virus to enter cells [12, 13]. Another study showed neuropathic corneal pain as a debilitating manifestation of the long-COVID syndrome [14].

We aim to provide physicians with a better understanding of the most common ocular symptoms, as appropriate recognition and handling of these symptoms should lead to a decrease in viral transmission and the number of infections. This review will discuss the current epidemiology of COVID-19, highlight the pathophysiology of how SARS-CoV-2 infects the ocular structures, identify the most common ocular complications, and finally list current therapeutics being used to treat COVID-19 infection. 

## Review

Methodology

The following search was conducted using PubMed to determine the extrapulmonary manifestations seen in COVID-19-positive patients. The initial query was through all fields containing keywords such as "COVID-19," "extrapulmonary," "symptoms," and "SARS-CoV-2." This search yielded potential extrapulmonary symptoms that have been recorded about the COVID-19 disease. Our primary search demonstrated the rise in the incidence of ocular manifestations seen in virus-positive patients. A secondary round of searches was conducted through PubMed, using keywords such as "ocular manifestations," "conjunctivitis," "optic manifestations," "episcleritis," and "kerato-conjunctivitis." These searches yielded an abundance of published literature regarding the ocular manifestations in those patients who contracted COVID-19. Around 233 articles from the aforementioned keywords were found. We excluded cases where the ophthalmic symptoms were not presented in the patient. We included articles covering five unique ophthalmic manifestations of COVID-19: conjunctivitis, episcleritis, central retinal artery occlusion, acute abducens nerve palsy, and ophthalmoparesis. We have compiled literature reviews, case reports, and meta-analyses to present the epidemiology, pathophysiology, and incidence of various COVID-19 ocular manifestations. Due to the recent nature of SARS-CoV-2, all articles published from 2020 to 2024 were considered as no prior literature existed. Further research into the risk factors and transmission should be conducted as a rising number of patients with ophthalmic symptoms become more prevalent.

Epidemiology

The most common clinical symptoms of COVID-19 include fever, cough, muscular soreness, and dyspnea; some atypical symptoms include diarrhea, nausea, and vomiting [15]. These nonspecific symptoms are commonly confounded by underlying medical conditions, as 25% of COVID-19-positive patients experience one or more of these symptoms [16]. Regarding severe COVID-19 manifestation in patients, a systematic review of 6,007 articles and 212 clinical studies found that underlying immunosuppression, diabetes, and malignancy were conditions that were highly associated with severe COVID-19 [17]. Individuals with the clinical features of diabetes, malignancy, and immunosuppression should be primarily targeted to prevent COVID-19 manifestation. As described by Guan et al. [18], COVID-19's reported characteristics and outcomes vary significantly among patients. Older patients were more susceptible to severe disease. A variety of symptoms were reported, from myalgia, fever, fatigue, and sputum being the most common to some patients reporting rash, hemoptysis, and conjunctival congestion as less common. The latter, conjunctival congestion, has been of particular interest. The paper by Nasiri et al. [19] pooled 38 studies with a total of 8,219 COVID-19 patients and described that the most commonly reported ocular symptoms included dry eye or foreign body sensation (16.0%), redness (13.3%), tearing (12.8%), and conjunctivitis (88.8%) as the most prevalent ocular signs. 

A variety of risk factors exist that vary from different categories such as comorbidities, demographics, and lifestyle. Both elderly age and the male gender are linked to higher severity [15]. Additionally, a study analyzing 10,926 COVID-19-related deaths concluded that both South Asian and Black patient populations were found to have an increased mortality risk compared with White patients [17]. A review of risk factors associated with COVID-19 determined that Black and other minority groups were disproportionately affected by an increased risk of hospitalization and mortality [16]. However, hypertension could be a confounding variable in the elderly population, and arterial hypertension was more frequently seen in patients with severe COVID-19 [16, 20]. Diabetes and obesity are other well-known risk factors for severe disease or death from COVID-19 [16, 21, 22]. A meta-analysis showed that COVID-19 patients with diabetes had a higher risk of severe disease as well as an increased ICU admission rate [20, 22]. Coronavirus studies have suggested that patients with HIV often have a reduced risk of both virus infection and severe disease course [23].

As the pandemic continues to progress, more information on the novel coronavirus is being gathered by physicians and scientists. Along with the pulmonary manifestations, multiple extrapulmonary symptoms are being recorded every day. There is a growing number of cases reported on patients suffering from ocular manifestations either as cardinal symptoms or as secondary symptoms of COVID-19 infection. A cross-sectional study of 535 COVID-19-positive patients was performed in Wuhan, China, to determine how many of them presented with ocular symptoms. Of the 535 surveyed, 5%, or 27 patients presented with conjunctival congestion. This study postulates that frequent hand-eye contact with patients may have been a direct cause of the congestion experienced. Further, this study advocates for patients to be screened for ophthalmic conditions, which may encompass the initial presentation for atypical COVID-19 cases [24]. Another cross-sectional, observational study is described in Abrishami et al. [25] in which a total of 142 patients were examined in Iran. Of those 142, 44 patients were noted as having conjunctival hyperemia, and 22 exhibited chemosis. The study indicated that conjunctival hyperemia was the most common symptom exhibited in all patients, while chemosis was the most common in those patients in the intensive care unit. Approximately half of all patients observed experienced some type of ocular manifestation [25]. Nasiri et al. [19] conducted a meta-analysis of previously published data which encompassed 8,219 COVID-19-positive patients. It was found that approximately 11.03% of patients presented with some type of ocular manifestation. Nasiri et al. [19] also reported that conjunctivitis had the highest rate among COVID-19 patients. This paper posits that approximately one out of every ten positive patients manifests some type of ocular symptoms.

Pathophysiology

SARS-CoV-2 is a Coronaviridae, a virus that is positive-sense, enveloped, single-stranded RNA. SARS-CoV-2 is also a member of the *Betacoronavirus *genus and the Orthocoronaviridae subfamily [26]. The virus shares a similar genome to previous coronaviruses: severe acute respiratory syndrome coronavirus 1 (SARS-CoV-1) and Middle Eastern respiratory syndrome coronavirus (MERS-CoV). COVID-19 is spread primarily by anthropophilic transmission via respiratory droplets. After contacting a person who is infected, where respiratory droplets can be inhaled, the virus can enter the upper respiratory tract and invade cells to replicate. Angiotensin-converting enzyme 2 receptor is the primary method to enter cells [11]. Spike (S) glycoproteins specifically attach to the ACE2 receptor, which is then cleaved using a protease, specifically transmembrane protease serine 2 (TMPRSS2), allowing the virus to enter the cell [27]. Once the virus enters the cell, it uncoats, allowing its genome to be both transcribed and subsequently translated [27]. ACE2 receptors are primarily located in the lung's type II alveolar pneumocytes, causing the predominance of pulmonary symptoms. After COVID-19 infection, ACE2 becomes downregulated leading to the production of reactive oxygen species (ROS), which further augments alveolar inflammation [28]. Zhou et al. [10] examined postmortem eyes and surgical specimens of COVID-19-positive patients. Immunohistochemical assays were performed on each specimen, revealing ACE2 receptors TMPRSS2, expressed in the conjunctiva, cornea, and limbus surface. The findings presented in this paper emphasize the importance of the eye as a reservoir for COVID-19 and how transmission can occur without proper eye and hand care [10].

The main route of transmission is through respiratory droplets that, when exposed to it, can travel through the respiratory tract. Hence, the global increase in facemask usage has been a critical element in reducing the spread of this virus. ACE2 receptors have been discovered as the primary cellular receptor primarily responsible for binding to the spike proteins found in the SARS-CoV-2 virus. ACE2 receptors have been found in both the respiratory mucosa and the small intestine. TMPRSS2 cleaves the spike proteins allowing for endosomal peptide fusion. Infective respiratory droplets containing the SARS-CoV-2 virus travel to the lungs, where the most significant number of ACE2 receptors are located. This is the accepted method by which SARS-CoV-2 can travel from person to person and cause infection [29]. Hence, the majority of COVID-19 infections lead to infections primarily affecting the lungs.

Chen et al. [24] state that transmission through the ocular mucosa is another viable form of transmission and that proper hand hygiene is another public health measure used to combat transmission of the virus. Zhou et al. [10] further add to the ocular transmission hypothesis with their discovery of ACE2 receptors found in the conjunctiva, cornea, and limbus surface. These ACE2 receptors serve as the direct entry point for the virus by binding to their spike proteins on the outer surface. Further endocytosis into the cells leads to viral RNA replication. Furthermore, A prospective interventional case series was conducted where conjunctival secretions were gathered twice every couple of days and sampled using a reverse transcription-polymerase chain reaction (RT-PCR). Although only one of the 30 patients sampled had positive RT-PCR results, there are several factors at play to consider. Adequate tear samples may not have been obtained, for example. Nevertheless, Xia et al. [30] speculate that SARS-CoV-2 may be detected in tears and conjunctival secretions.

**Figure 1:**
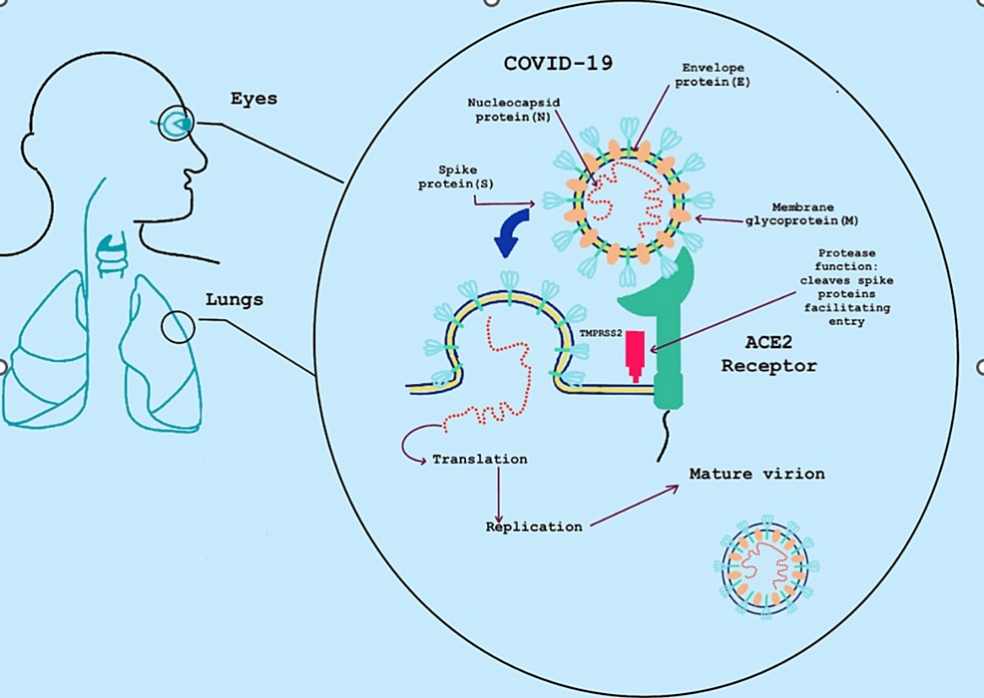
Depicts the pathophysiology of the coronavirus binding to the ACE2 receptor, which has been known to be prevalent in both the eyes and lungs, leading to pulmonary and potentially ophthalmic manifestations ACE2: Angiotensin-converting enzyme 2

After the entry of the SARS-CoV-2 into cells, an abundance of pro-inflammatory cytokines is released such as interleukin 6 (IL-6), IL-1β, IL-8, IL-12, macrophage inflammatory protein 1A (MIP1A), interferon-gamma-inducible protein (IP10), monocyte chemoattractant protein 1 (MCP1), tumor necrosis factor-α (TNF-α), and IP10 [29]. The release of these pro-inflammatory markers could be directly causing damage to the ocular mucosa leading to episcleritis or conjunctivitis, as seen in those COVID-19-positive patients presenting with ophthalmic manifestations of symptoms. Refer to Figure 1.

Reported ocular manifestations

Conjunctivitis

Conjunctivitis is characterized by inflammation or infection of the transparent membrane (conjunctiva). Conjunctivitis is the most common ocular manifestation of COVID-19, with approximately one in every ten patients presenting with conjunctivitis symptoms associated with COVID-19 [14]. Güemes-Villahoz et al. [31] conducted a cross-sectional study analyzing conjunctivitis in COVID-19 patients. It has been reiterated that conjunctivitis is an uncommon manifestation of COVID-19, but it is the most common ocular manifestation. The study calculated the prevalence of conjunctivitis among hospitalized patients to be 11.6%. A recent case report published by Cheema et al. [32] describes keratoconjunctivitis as one of the initial presenting symptoms of COVID-19. The patient has had mild respiratory symptoms, no fever, and initially presented to the ophthalmology department with keratoconjunctivitis. Three days after his diagnosis of keratoconjunctivitis, the patient tested positive for the SARS-CoV-2 virus [32]. Retrospective testing of the eyes was performed and yielded that SARS-Cov-2 levels were weakly positive [32]. Guo et al. [20] present a case of a 53-year-old male who presented with viral conjunctivitis in his right eye ten days after his COVID-19 diagnosis. Although no COVID-19 was detected in conjunctival secretions, increased IL-6 was noted to be increased. These findings potentiate the involvement of cytokine surge in the development of conjunctivitis. Patients with red eyes, and respiratory symptoms, and who have recently traveled to a COVID-19 outbreak location must be treated with additional precaution as they have a higher risk of disease.

Episcleritis

Episcleritis is a benign, inflammatory disease affecting the episcleral tissue and is not commonly considered in the manifestation of COVID-19 [33]. Méndez Mangana et al. [33] reported the first case of episcleritis in a COVID-19-positive patient. The clinical presentation of the case they reported met the criteria for acute nodular episcleritis, which developed in the patient seven days after the onset of COVID-19 infection. They described a pathophysiological theory that includes immunovascular factors and coagulation factors as possible relationships between COVID-19 and episcleritis. This patient was treated with artificial tears, and fluorometholone was prescribed to take five times a day for three days. Another case of episcleritis was described by Otaif et al. [34], in which a 29-year-old male patient developed a mild viral infection three days after the ocular signs of episcleritis. Usually, episcleritis is idiopathic and benign; however, an association between episcleritis and systemic disease has been found in less than one-third of patients. Episcleritis has also been found as an ocular manifestation in herpes zoster, hepatitis C, Ebola virus, and possibly COVID-19 [34].

Ophthalmoparesis

Ophthalmoparesis is a weakness or paralysis of any of the extraocular muscles that garner movement of the eye. They include the superior, medial, inferior, and lateral rectus and the superior and inferior obliques. The correlation between SARS-CoV-2 and opthalmoparesis was discussed in a study by Dinkin et al. [35] in which two cases involving cranial nerve palsy were presented after the positive COVID-19 test results. Both cases involved an onset of fever and cough before the onset of the cranial nerve palsies. In case 1, the patient also experienced neurological symptoms like leg paresthesia secondary to myalgias. The onset of cranial nerve palsy in the first case was notable for loss of control of the lateral rectus muscle in the left eye as there was a loss of left eye abduction and depression. These symptoms worsened during their inpatient stay before recovering after IV immunoglobulin administration. It was determined that Miller Fisher syndrome resulting from COVID-19 infection was the cause of the abducens nerve palsy in this case. Dinkin et al. [35] studied another case, a 71-year-old woman who experienced similar diplopia and loss of abduction of her right eye with a magnetic resonance imaging (MRI) showing signs of optic nerve inflammation resulting from COVID-19 infection. In both cases, abnormal ophthalmic findings were found within a week of the onset of COVID-19-related symptoms and quickly resolved once the body had cleared the virus. Dinkin et al. [35] also highlighted the occurrence of CNS symptoms in patients correlated with lymphopenia, something that is seen on a more widespread basis in Wuhan, China, the original epicenter of the virus.

Central Retinal Artery Occlusion (CRAO)

CRAO is a serious disease of the eye in which the main artery supplying the retina is blocked due to an atherosclerotic plaque. These plaques are usually the result of an atherosclerotic plaque in another artery that becomes embolized and travels into the retinal artery. Plaques then lodge, leading to partial or full stenosis of the blood vessel to the retina. A case of CRAO secondary to COVID-19 was studied by Acharya et al. [36]. A 60-year-old patient presents with acute exacerbation of symptoms due to COVID-19 and, while in an inpatient setting, complains of a sudden loss of vision in his left eye. The ophthalmology department was able to confirm a left CRAO, and the lab tests confirmed the presence of inflammation (elevated IL-6) and elevated clotting (D-dimer), which pointed to CRAO occurring after the patient's COVID-19 diagnosis. Acharya et al. [36] highlight a case as the first hypercoagulable, hyperinflammatory response to COVID-19 in the eye that has been documented. It also showed the heightened need for physicians to be wary of ischemic events occurring anywhere in the body in the face of COVID-19 infections. Treating CRAO is complex, with most treatment options not improving outcomes and the loss of eyesight being a typical imminent prognosis.

Acute Abducens Nerve Palsy

The abducens nerve is the sixth cranial nerve that provides innervation to the lateral rectus eye muscle. The primary function of the lateral rectus muscle is to help abduct the eyeball. Abducens nerve palsy is the most common motor ocular muscle paralysis in adults and will lead to esotropia. Falcone et al. [37] presented a case of acute abducens nerve palsy in a 32-year-old male following SARS-CoV-2 infection. Following three days of worsening upper respiratory-like symptoms, the patient developed acute, binocular, horizontal diplopia upon waking one morning. The patient's prognosis worsened, and he was hospitalized for hypoxemic respiratory failure. At the time, he was prescribed hydroxychloroquine with supportive oxygen for five days, and he quickly recovered. Following recovery, the patient underwent an ophthalmic examination revealing left eye esotropia. Hyperintense left lateral rectus muscle on T2-weighted MRI confirmed atrophy of the muscle, which has been consistent with acute abducens nerve palsy. Falcone et al. [37] hypothesized that the patient had a direct or an indirect virally mediated insult that occurred in the abducens nerve leading to the atrophy and subsequent palsy. Refer to Table 1.

**Table 1:** This table summarizes each of the individual case reports. The case published, patients age, and reported symptoms are highlighted. Additionally, an explanation of each treatment method used at the time for every specific ocular manifestation is included. CRAO: Central retinal artery occlusion; TID: ter in die (three times a day); QID: quarter in die (four times a day); mg: milligrams; kg: kilograms; PO: per orem

Case	Demographic	Clinical manifestations	Treatment (if any)
Gao et al. [16] (keratoconjunctivitis)	53-year-old male	Fever, cough, edema, redness in the eye, viscous watery secretions, and bulbar conjunctival hyperemia	0.1% fluorometholone (drops, TID), levofloxacin hydrochloride (drops, TID), 0.1% sodium hyaluronate (drops, TID)
Cheema et al. [32] (keratoconjunctivitis)	29-year-old female	Photophobia, clear watery discharge, and conjunctivitis in her right eye for the past 24 hours. Right, tender, periauricular lymph node.	Moxifloxacin 1 drop QID and oral valacyclovir 500 mg PO TID.
Méndez Mangana et al. [33] (episcleritis)	31-year-old female	Myalgia, cough, ageusia, anosmia, foreign‐body sensation in the eye, epiphora, photophobia without impaired visual acuity, and referring red eye.	Artificial tears on-demand and fluorometholone 5x/day for three days.
Montesel et al. [38] (central retinal artery occlusion)	59-year-old male	Fever, dyspnea, dry cough, sudden onset of painless loss of vision and nonreactive mydriasis in the left eye, and dilated fundus ophthalmoscopy revealed retinal whitening in the macular region with loss of the physiological macular reflex and peripheral areas of retinal pigment epithelium hyperpigmentation and a presence of severe arterial narrowing.	Lopinavir/ritonavir association (200 and 50 mg, respectively) twice per day, hydroxychloroquine 400 mg twice per day, and a single intravenous dose of tocilizumab 800 mg.
Acharya et al. [36] (central retinal artery occlusion)	60-year-old male	Cough, dyspnea, fever, sudden onset of painless loss of vision in the right eye, cherry-red spot on the right optic nerve, pupils unresponsive to light, and absent accommodation reflex.	No treatment is available for CRAO yet. Azithromycin, hydroxychloroquine, and tocilizumab for typical Covid-19 symptoms.
Dinkin et al. [35] (opthalmoparesis from cranial nerve palsy)	36-year-old male	Left ptosis, diplopia, bilateral distal leg paresthesia, complete loss of depression and horizontal eye movements on the left, and loss of abduction on the right eye.	Hydroxychloroquine for COVID-19 (600 mg twice a day for one day, followed by 400 mg daily for four days). IV immunoglobulin (2g/kg over three days).
Dinkin et al. [35] (opthalmoparesis from cranial nerve palsy)	71-year-old female	Cough, fever, and painless diplopia on waking two days before and loss of abduction in the right eye.	Hydroxychloroquine for COVID-19 (600 mg twice a day for one day, followed by 400 mg daily for four days)
Falcone et al. [37] (acute abducens nerve palsy)	32-year-old male	Binocular, acute, horizontal diplopia on waking following three days of upper respiratory illness symptoms.	Supplemental oxygen and a five-day course of hydroxychloroquine

COVID-19 therapeutics review

Due to the novelty of COVID-19, there have yet to be any curative therapies identified. Supportive care becomes the mainstay therapy option. Supportive care can differ depending on the degree of illness severity, but it most often includes but is not limited to intravenous fluids, supplemental oxygen, and fever reducers. While no antiviral therapies exist, multiple existing antiviral medications already in use have been used with great efficacy to treat severe infections. Currently, there is no cure for ocular COVID-19 infection, but the current regimen calls for symptomatic treatment of ocular manifestations.

Early, both chloroquine and hydroxychloroquine emerged as potential therapies for severe hospitalized COVID-19. The United States Food and Drug Administration (USFDA) approved an emergency use authorization for both drugs due to some initial reports of decreased infection severity. Chloroquine and hydroxychloroquine are traditionally used as drugs to treat malaria, blocking viral endosomal-mediated fusion [38]. Additionally, they have been used in many autoimmune conditions. Both drugs have exhibited some type of anti-inflammatory effect. However, conflicting case reports have since been reported, with many side effects noted for both hydroxychloroquine and chloroquine. Prolonged QT interval as well as retinal damage and ocular toxicity are two dangerous adverse effects reported [39]. This information is imperative as those patients with ocular symptoms should not be prescribed chloroquine or hydroxychloroquine as they can worsen ocular symptoms. The FDA has revoked the emergency use authorization as the risks seem to outweigh the benefits of these medications.

Remdesivir is an antiviral drug that was first developed for use against the Ebola virus. It is an adenosine analog prodrug, which inhibits viral RNA transcription [39]. Remdesivir is the antiviral that has shown the greatest effectiveness in leading to quicker recovery times. Animal studies using mice infected with SARS-CoV, treated with remdesivir, exhibited both reduced lung pathology and viral burden. It was reported in February 2020 that remdesivir exhibited increased efficacy in SARS-CoV in vitro. Successful animal trials have led to increased clinical application of intravenous remdesivir in severe SARS-CoV-2-positive patients. Within 24 hours of intravenous delivery, patients were weaned off the nasal cannula, became afebrile, and chest rales resolved [39]. As it stands today, remdesivir has been recommended by the National Institutes of Health (NIH) for severe hospitalized COVID-19 cases as defined by specific oxygenation requirements [39]. However, there does not seem to be any evidence indicating that remdesivir leads to decreased mortality rates.

Lopinavir/ritonavir (abbreviated as LPV/r) is a combination product used to treat human immunodeficiency virus (HIV) infection by potentially slowing disease progression. The method of action of lopinavir occurs by blocking viral protease and preventing GAG-POL polyprotein cleavage. Lopinavir is then metabolized by cytochrome 3A (CYP3A). Ritonavir, however, acts as a cytochrome P450 (CYP450) inhibitor and increases the concentration of lopinavir in the plasma, leading to a greater duration of action and effect. Lopinavir demonstrated use in an in vitro cytopathic effect against SARS-CoV at 4 μg/mL [39].

Cao et al. [40] showed a trial of the lopinavir/ritonavir combination product was conducted in hospitalized patients with serious COVID-19 complications. Mortality was the same in the LPV/r group vs. the control group. This study concluded that there was no benefit for LPV/r over standard treatment; however, the median time for improvement was reduced by one day or so. Further clinical trials should be conducted to determine the clear efficacy or lack of COVID-19 infection.

At the time of the reported cases, only hydroxychloroquine was available and was used to manage severe COVID-19 infections. There is no indication that it was effective at reducing the frequency or even in treating ocular symptoms from COVID-19.

## Conclusions

We have discovered that the ocular system is particularly susceptible to the COVID-19 virus with numerous symptoms having been reported across the globe. ACE2 receptors are present in the eye, allowing for easy transmission of the virus into the conjunctiva. Conjunctivitis is by far the most common ocular symptom experienced by infected individuals. However, more serious complications such as CRAO and cranial nerve palsies have also been identified. Rapid diagnosis and swift treatment of these complications should be paramount to prevent any permanent damage to those patients. Current treatment regimens should include symptomatic treatment as no cures for COVID-19 currently exist. However, in December 2021, the FDA approved the Paxlovid (nirmatrelvir/ritonavir) medication for those patients with mild to moderate COVID-19 infection who may have a risk of progressing to serious disease. These medications halt viral replication and reduce viral load which could improve the inflammatory burden the virus causes throughout the body. This new medication may help treat COVID-19-positive patients with ophthalmic symptoms. As the number of COVID-19 cases with ophthalmic infection continues to rise, further research must be conducted into the specific pathophysiology of ocular manifestations. Physicians should be informed on how to treat these symptoms, should they come into contact with those patients presenting with ophthalmic manifestations of COVID-19.
